# Complete genome sequence of *Paludibacter propionicigenes* type strain (WB4^T^)

**DOI:** 10.4056/sigs.1503846

**Published:** 2011-02-20

**Authors:** Sabine Gronow, Christine Munk, Alla Lapidus, Matt Nolan, Susan Lucas, Nancy Hammon, Shweta Deshpande, Jan-Fang Cheng, Roxane Tapia, Cliff Han, Lynne Goodwin, Sam Pitluck, Konstantinos Liolios, Natalia Ivanova, Konstantinos Mavromatis, Natalia Mikhailova, Amrita Pati, Amy Chen, Krishna Palaniappan, Miriam Land, Loren Hauser, Yun-Juan Chang, Cynthia D. Jeffries, Evelyne Brambilla, Manfred Rohde, Markus Göker, John C. Detter, Tanja Woyke, James Bristow, Jonathan A. Eisen, Victor Markowitz, Philip Hugenholtz, Nikos C. Kyrpides, Hans-Peter Klenk

**Affiliations:** 1 DSMZ - German Collection of Microorganisms and Cell Cultures GmbH, Braunschweig, Germany; 2 DOE Joint Genome Institute, Walnut Creek, California, USA; 3 Los Alamos National Laboratory, Bioscience Division, Los Alamos, New Mexico, USA; 4 Biological Data Management and Technology Center, Lawrence Berkeley National Laboratory, Berkeley, California, USA; 5 Oak Ridge National Laboratory, Oak Ridge, Tennessee, USA; 6 HZI – Helmholtz Centre for Infection Research, Braunschweig, Germany; 7 University of California Davis Genome Center, Davis, California, USA; 8 Australian Centre for Ecogenomics, School of Chemistry and Molecular Biosciences,The University of Queensland, Brisbane, Australia

**Keywords:** strictly anaerobic, nonmotile, Gram-negative, anoxic rice-field soil, mesophilic, chemoorganotrophic, *Porphyromonadaceae*, GEBA

## Abstract

*Paludibacter propionicigenes* Ueki *et al*. 2006 is the type species of the genus *Paludibacter*, which belongs to the family *Porphyromonadaceae*. The species is of interest because of the position it occupies in the tree of life where it can be found in close proximity to members of the genus *Dysgonomonas*. This is the first completed genome sequence of a member of the genus *Paludibacter* and the third sequence from the family *Porphyromonadaceae*. The 3,685,504 bp long genome with its 3,054 protein-coding and 64 RNA genes consists of one circular chromosome and is a part of the *** G****enomic* *** E****ncyclopedia of* *** B****acteria and* *** A****rchaea * project.

## Introduction

Strain WB4^T^ (= DSM 17365 = CCUG 53888 = JCM 13257) is the type strain of *P. propionicigenes*, which is the type species of the genus *Paludibacter* [1,2]. Currently, there is only one species placed in the genus *Paludibacter* [1]. The generic name derives from the Latin noun *palus* –*udis* meaning swamp or marsh and the Neo-Latin word *bacter* meaning *a rod*, referring to a rod living in swamps [2]. The species epithet is derived from the Neo-Latin word *acidum propionicum* meaning *propionic acid* and the Greek verb *gennao* meaning *to produce*, referring to the metabolic property of the species [2]. *P. propionicigenes* strain WB4^T^ was isolated together with a number of other strains from rice plant residues in an anoxic rice-field soil in Yamagata, Japan, and described for the first time by Akasaka *et al*. in 2003 [3]. In 2006 the species was formally described by Ueki *et al*. and the genus *Paludibacter* was introduced [2]. No further isolates have been obtained for *P. propionicigenes*, however, cultivation-independent 16S rRNA-dependent molecular investigations showed the presence of *P. propionicigenes* in the rumen of sheep [4]. Here we present a summary classification and a set of features for *P. propionicigenes* WB4^T^, together with the description of the complete genomic sequencing and annotation.

## Classification and features

A representative genomic 16S rRNA sequence of strain WB4^T^ was compared using NCBI BLAST under default values (e.g., considering only the best 250 hits) with the most recent release of the Greengenes database [5] and the relative frequencies, of taxa and keywords (reduced to their stems [6]) were determined, weighted by BLAST scores. The most frequently occurring genus was *Dysgonomonas* (100%) (8 hits in total). Among all other species, the one yielding the highest score was *Dysgonomonas capnocytophagoides*, which corresponded to an identity of 91.9% and a HSP coverage of 83.6%. The highest-scoring environmental sequence was AY212569 ('water 10 m downstream manure clone 118ds10'), which showed an identity of 99.6% and a HSP coverage of 100.1%. The five most frequent keywords within the labels of environmental samples which yielded hits were 'digest' (11.7%), 'anaerob' (6.2%), 'sludge' (6.1%), 'wastewater' (6.0%) and 'mesophile' (5.9%) (241 hits in total). The single most frequent keyword within the labels of environmental samples which yielded hits of a higher score than the highest scoring species was 'downstream/manure/water' (33.3%) (1 hit in total).

**Figure 1:**
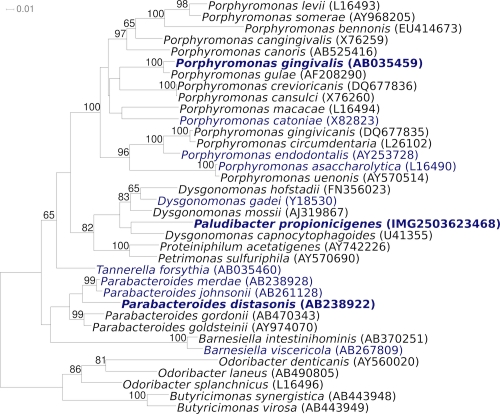
Phylogenetic tree highlighting the position of *P. propionicigenes* relative to the other type strains within the family *Porphyromonadaceae*. The tree was inferred from 1,400 aligned characters [7,8] of the 16S rRNA gene sequence under the maximum likelihood criterion [9] and rooted in accordance with the current taxonomy. The branches are scaled in terms of the expected number of substitutions per site. Numbers above branches are support values from 300 bootstrap replicates [10] if larger than 60%. Lineages with type strain genome sequencing projects registered in GOLD [11] are shown in blue, published genomes in bold [12,13].

Figure 1 shows the phylogenetic neighborhood of *P. propionicigenes* WB4^T^ in a 16S rRNA based tree. The three identical 16S rRNA sequences in the genome differ by one nucleotide from the previously published 16S rRNA sequence (AB078842).

**Figure 2:**
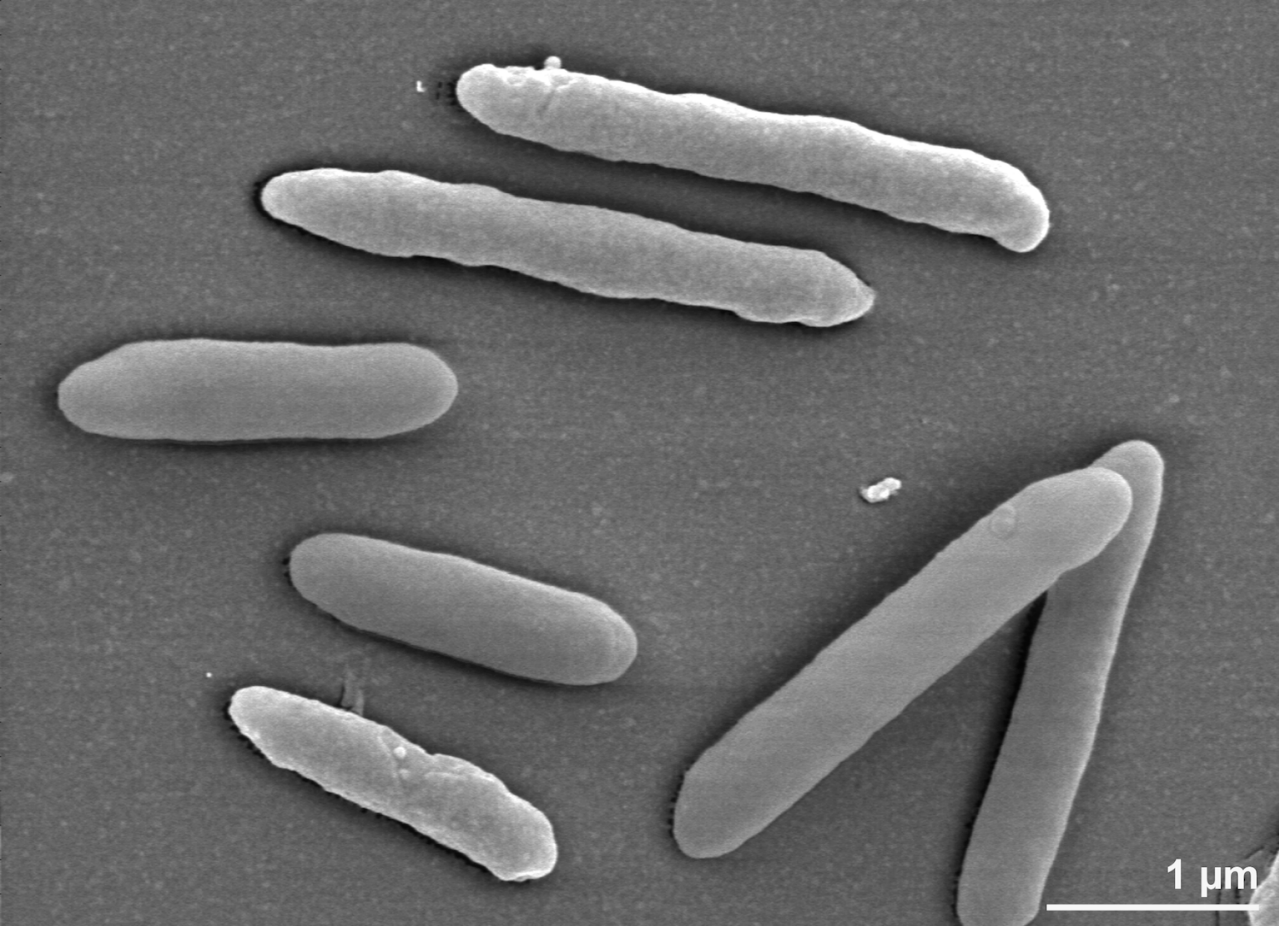
Scanning electron micrograph of *P. propionicigenes* WB4^T^

The cells of *P. propionicigenes* are generally rod-shaped (0.5-0.6 μm × 1.3-1.7 µm) with ends that are round or slightly tapered [2]. Elongated cells can also be seen, either as single cells or in short chains (Figure 2). *P. propionicigenes* is a Gram-negative and non spore-forming bacterium (Table 1). The organism is described to be nonmotile; only eight genes associated with motility were identified in the genome. The organism is strictly anaerobic and chemoorganotrophic [2,3]. The temperature range for growth is between 15°C and 35°C, with an optimum at 30°C [2]. The organism does not grow at 37°C [2]. The pH range for growth is 5.0-7.6 with an optimum at pH 6.6 [2]. NaCl concentrations from 0-0.5% (w/v) are tolerated. *P. propionicigenes* is able to utilize arabinose, glucose, fructose, xylose, cellobiose, galactose, mannose, maltose, melibiose, glycogen and soluble starch as growth substrates [2]. The organism does not utilize ribose, lactose, sucrose, melezitose, raffinose, sorbose, rhamnose, trehalose, cellulose, xylan, salicin, dulcitol, inositol, mannitol, sorbitol, ethanol, glycerol, fumarate, malate, lactate, succinate or pyruvate [2]. Glucose is fermented to propionate and acetate in a molar ratio of 2:1 as major products and succinate as a minor product [2]. The organism does not reduce nitrate, it does not hydrolyze gelatin or urea and does not produce indole or hydrogen sulfide [2]. *P. propionicigenes* does not grow in the presence of bile salts. Catalase and oxidase are not present in the organism [2].

**Table 1:** Classification and general features of *P. propionicigenes* WB4^T^ according to the MIGS recommendations [14].

MIGS ID	Property	Term	Evidence code
	Current classification	Domain *Bacteria*	TAS [15]
		Phylum *Bacteroidetes*	TAS [16]
		Class *Bacteroidia*	TAS [16,17]
		Order *Bacteroidales*	TAS [16]
		Family *Porphyromonadaceae*	TAS [16]
		Genus *Paludibacter*	TAS [2]
		Species *Paludibacter propionicigenes*	TAS [2]
		Type strain WB4	TAS [2]
	Gram stain	negative	TAS [3]
	Cell shape	rod-shaped	TAS [3]
	Motility	non-motile	TAS [2]
	Sporulation	none	TAS [3]
	Temperature range	15°C to 35°C	TAS [3]
	Optimum temperature	30°C	TAS [2]
	Salinity	normal	NAS
MIGS-22	Oxygen requirement	strictly anaerobic	TAS [3]
	Carbon source	carbohydrates	TAS [3]
	Energy source	chemoorganotroph	TAS [3]
MIGS-6	Habitat	soil	TAS [3]
MIGS-15	Biotic relationship	free-living	NAS
MIGS-14	Pathogenicity	none	NAS
	Biosafety level	1	TAS [18]
	Isolation	rice plant residue in anoxic rice-field soil	TAS [3]
MIGS-4	Geographic location	Yamagata, Japan	TAS [3]
MIGS-5	Sample collection time	1994	TAS [3]
MIGS-4.1	Latitude	38.25	NAS
MIGS-4.2	Longitude	140.34	NAS
MIGS-4.3	Depth	not reported	
MIGS-4.4	Altitude	not reported	

Evidence codes - IDA: Inferred from Direct Assay (first time in publication); TAS: Traceable Author Statement (i.e., a direct report exists in the literature); NAS: Non-traceable Author Statement (i.e., not directly observed for the living, isolated sample, but based on a generally accepted property for the species, or anecdotal evidence). These evidence codes are from of the Gene Ontology project [19]. If the evidence code is IDA, then the property was directly observed by one of the authors or an expert mentioned in the acknowledgements.

### Chemotaxonomy

Little chemotaxonomic data are available for strain WB4^T^. Only the fatty acid composition has been elucidated. The major fatty acids found were *anteiso-*C_15:0_ (30.8%), C_15:0_ (19.0%) and 3-hydroxy *anteiso-*C_17:0_ (17.9%) [2]. Also, *iso-*C_17:0 3-OH_ (6.2%) and C_16:0_ (4.9%) were detected in intermediate amounts whereas *iso-*C_15:0 3-OH_, *iso-*C_16:0 3-OH_, C_15:0 3-OH_, C_16:03-OH_, *iso*-C_15:0_, C_14:0_, C_16:0_, and C_18:0_ were present in minor amounts (1% to 5% of the total fatty acids). Unsaturated fatty acids were not detected [2].

## Genome sequencing and annotation

### Genome project history

This organism was selected for sequencing on the basis of its phylogenetic position [20], and is part of the *** G****enomic* *** E****ncyclopedia of* *** B****acteria and* *** A****rchaea * project [21]. The genome project is deposited in the Genomes OnLine Database [11] and the complete genome sequence is deposited in GenBank. Sequencing, finishing and annotation were performed by the DOE Joint Genome Institute (JGI). A summary of the project information is shown in Table 2.

**Table 2:** Genome sequencing project information

**MIGS ID**	**Property**	**Term**
MIGS-31	Finishing quality	Finished
MIGS-28	Libraries used	Three genomic libraries: one 454 pyrosequence standard library, one 454 PE library (9 kb insert size), one Illumina library
MIGS-29	Sequencing platforms	Illumina GAii, 454 GS FLX Titanium
MIGS-31.2	Sequencing coverage	337.6 × Illumina; 28.1 × pyrosequence
MIGS-30	Assemblers	Newbler version 2 2.3-PreRelease-10-21-2009-gcc-4.1.2-threads, Velvet, phrap
MIGS-32	Gene calling method	Prodigal 1.4, GenePRIMP
	INSDC ID	CP002345
	Genbank Date of Release	December 2, 2010
	GOLD ID	Gc01549
	NCBI project ID	694427
	Database: IMG-GEBA	2503538024
MIGS-13	Source material identifier	DSM 17365
	Project relevance	Tree of Life, GEBA

### Growth conditions and DNA isolation

*P. propionicigenes* WB4^T^, DSM 17365, was grown anaerobically in DSMZ medium 104 [22] at 30°C. DNA was isolated from 0.5-1 g of cell paste using a MasterPure Gram-positive DNA purification kit (Epicentre MGP04100) following the standard protocol as recommended by the manufacturer, with modification st/DL for cell lysis as described in Wu *et al*. [21].

### Genome sequencing and assembly

The genome was sequenced using a combination of Illumina and 454 sequencing platforms. All general aspects of library construction and sequencing can be found at the JGI website [23]. Pyrosequencing reads were assembled using the Newbler assembler version 2.3-PreRelease-10-21-2009-gcc-4.1.2-threads (Roche). The initial Newbler assembly consisting of 26 contigs in one scaffold which was converted into a phrap assembly by [24] making fake reads from the consensus, to collect the read pairs in the 454 paired end library. Illumina GAii sequencing data (967 Mb) was assembled with Velvet [25] and the consensus sequences were shredded into 1.5 kb overlapped fake reads and assembled together with the 454 data. The 454 draft assembly was based on 93.4 Mb 454 draft data and all of the 454 paired end data. Newbler parameters are -consed -a 50 -l 350 -g -m -ml 20. The Phred/Phrap/Consed software package  was used for sequence assembly and quality assessment in the subsequent finishing process. After the shotgun stage, reads were assembled with parallel phrap (High Performance Software, LLC). Possible mis-assemblies were corrected with gapResolution [23], Dupfinisher, or sequencing cloned bridging PCR fragments with subcloning or transposon bombing (Epicentre Biotechnologies, Madison, WI) [26]. Gaps between contigs were closed by editing in Consed, by PCR and by Bubble PCR primer walks (J.-F.Chang, unpublished). A total of 124 additional reactions and one shatter library were necessary to close the gaps and to raise the quality of the finished sequence. Illumina reads were also used to correct potential base errors and increase consensus quality using a software Polisher developed at JGI [27]. The error rate of the completed genome sequence is less than 1 in 100,000. Together, the combination of the Illumina and 454 sequencing platforms provided 365.7 × coverage of the genome. The final assembly contained 333,397 pyrosequence and 34,564,373 Illumina reads.

### Genome annotation

Genes were identified using Prodigal [28] as part of the Oak Ridge National Laboratory genome annotation pipeline, followed by a round of manual curation using the JGI GenePRIMP pipeline [29]. The predicted CDSs were translated and used to search the National Center for Biotechnology Information (NCBI) nonredundant database, UniProt, TIGR-Fam, Pfam, PRIAM, KEGG, COG, and InterPro databases. Additional gene prediction analysis and functional annotation was performed within the Integrated Microbial Genomes - Expert Review (IMG-ER) platform [30].

## Genome properties

**Figure 3:**
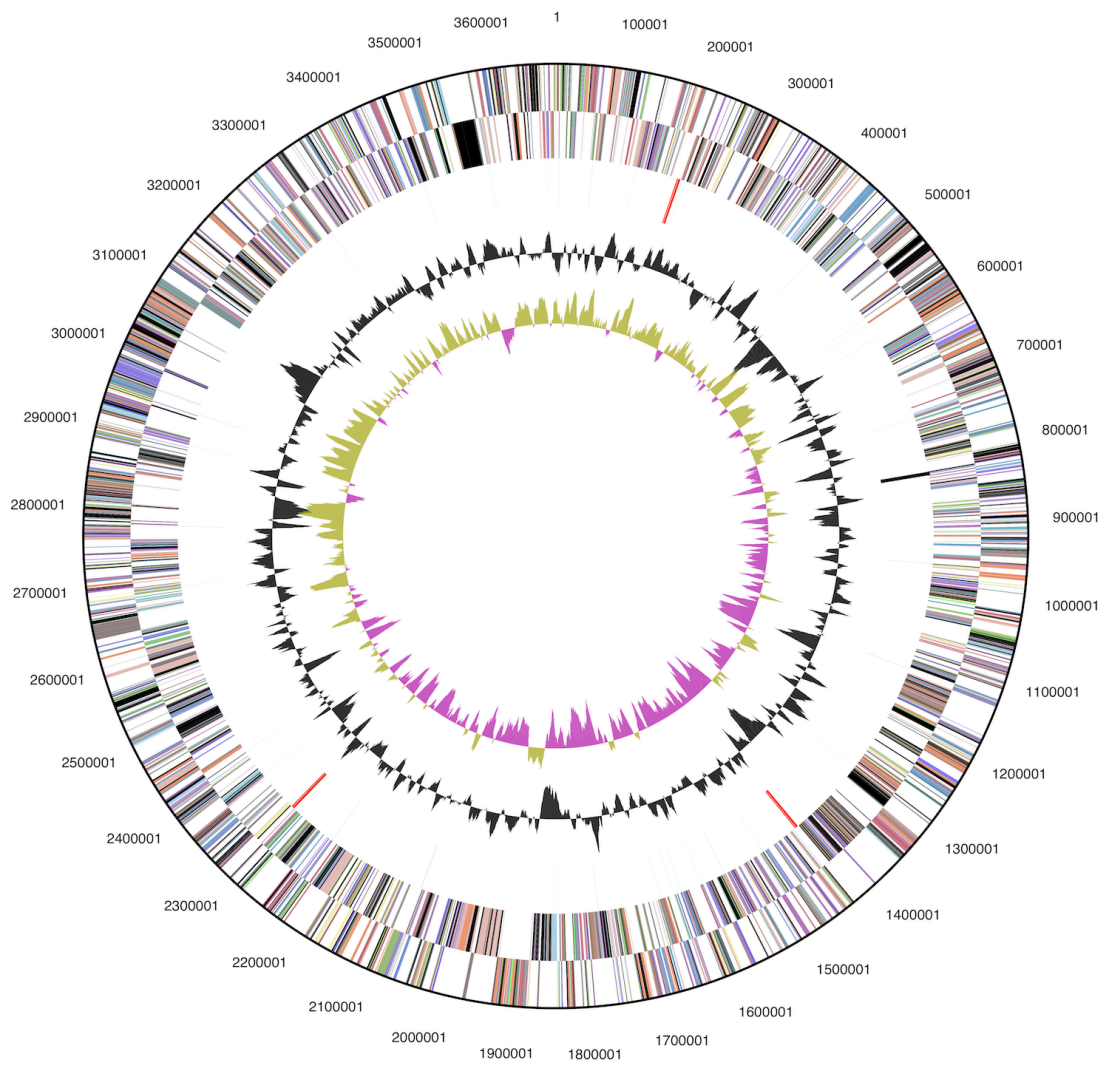
Graphical circular map of the chromosome. From outside to the center: Genes on forward strand (color by COG categories), Genes on reverse strand (color by COG categories), RNA genes (tRNAs green, rRNAs red, other RNAs black), GC content, GC skew.

The genome consists of a 3,685,504 bp long chromosome with a GC content of 38.9% (Table 3 and Figure 3). Of the 3,118 genes predicted, 3,054 were protein-coding genes, and 64 RNAs; 34 pseudogenes were also identified. The majority of the protein-coding genes (65.8%) were assigned with a putative function while the remaining ones were annotated as hypothetical proteins. The distribution of genes into COGs functional categories is presented in Table 4.

**Table 3:** Genome Statistics

**Attribute**	**Value**	**% of Total**
Genome size (bp)	3,685,504	100.00%
DNA coding region (bp)	3,225,817	87.53%
DNA G+C content (bp)	1,432,064	38.86%
Number of replicons	1	
Extrachromosomal elements	0	
Total genes	3,118	100.00%
RNA genes	64	2.05%
rRNA operons	3	
Protein-coding genes	3,054	97.95%
Pseudo genes	34	1.09%
Genes with function prediction	2,051	65.78%
Genes in paralog clusters	325	10.42%
Genes assigned to COGs	2,005	64.30%
Genes assigned Pfam domains	2,205	70.72%
Genes with signal peptides	843	27.04%
Genes with transmembrane helices	784	25.14%
CRISPR repeats	2	

**Table 4:** Number of genes associated with the general COG functional categories

**Code**	**value**	**%age**	**Description**
J	149	6.8	Translation, ribosomal structure and biogenesis
A	0	0	RNA processing and modification
K	136	6.2	Transcription
L	101	4.6	Replication, recombination and repair
B	0	0	Chromatin structure and dynamics
D	22	1.0	Cell cycle control, cell division, chromosome partitioning
Y	0	0	Nuclear structure
V	48	2.2	Defense mechanisms
T	99	4.5	Signal transduction mechanisms
M	232	10.6	Cell wall/membrane/envelope biogenesis
N	8	0.4	Cell motility
Z	0	0	Cytoskeleton
W	0	0	Extracellular structures
U	40	1.8	Intracellular trafficking, secretion, and vesicular transport
O	80	3.7	Posttranslational modification, protein turnover, chaperones
C	108	5.0	Energy production and conversion
G	172	7.9	Carbohydrate transport and metabolism
E	166	7.6	Amino acid transport and metabolism
F	61	2.8	Nucleotide transport and metabolism
H	128	5.9	Coenzyme transport and metabolism
I	67	3.1	Lipid transport and metabolism
P	131	6.0	Inorganic ion transport and metabolism
Q	24	1.1	Secondary metabolites biosynthesis, transport and catabolism
R	256	11.7	General function prediction only
S	153	7.0	Function unknown
-	1,113	35.7	Not in COGs
